# Quercetin Attenuates Podocyte Apoptosis of Diabetic Nephropathy Through Targeting EGFR Signaling

**DOI:** 10.3389/fphar.2021.792777

**Published:** 2022-01-05

**Authors:** Yiqi Liu, Yuan Li, Liu Xu, Jiasen Shi, Xiujuan Yu, Xue Wang, Xizhi Li, Hong Jiang, Tingting Yang, Xiaoxing Yin, Lei Du, Qian Lu

**Affiliations:** Jiangsu Key Laboratory of New Drug Research and Clinical Pharmacy, Xuzhou Medical University, Xuzhou, China

**Keywords:** quercetin, diabetic nephropathy, network pharmacology, podocyte apoptosis, EGFR

## Abstract

Podocytes injury is one of the leading causes of proteinuria in patients with diabetic nephropathy (DN), and is accompanied by podocytes apoptosis and the reduction of podocyte markers such as synaptopodin and nephrin. Therefore, attenuation of podocyte apoptosis is considered as an effective strategy to prevent the proteinuria in DN. In this study, we evaluated the anti-podocyte-apoptosis effect of quercetin which is a flavonol compound possessing an important role in prevention and treatment of DN and verified the effect by using *db/db* mice and high glucose (HG)-induced mouse podocytes (MPs). The results show that administration of quercetin attenuated the level of podocyte apoptosis by decreasing the expression of pro-apoptotic protein Bax, cleaved caspase 3 and increasing the expression of anti-apoptotic protein Bcl-2 in the *db/db* mice and HG-induced MPs. Furthermore, epidermal growth factor receptor (EGFR) was predicted to be the potential physiological target of quercetin by network pharmacology. *In vitro* and vivo experiments confirmed that quercetin inhibited activation of the EGFR signaling pathway by decreasing phosphorylation of EGFR and ERK1/2. Taken together, this study demonstrates that quercetin attenuated podocyte apoptosis through inhibiting EGFR signaling pathway, which provided a novel approach for further research of the mechanism of quercetin in the treatment of DN.

## Introduction

Diabetes is one of the fastest growing chronic diseases worldwide, which leads to devastating macrovascular and microvascular complications. Diabetic nephropathy (DN) is one of the most serious complications of diabetes (Gnudi et al., 2016). Glomerular hyperfiltration and proteinuria are the early clinical manifestations of DN. The pathological features of proteinuria formation are mesangial dilatation, endothelial cell degeneration and podocyte injury (Chen et al., 2015; Du et al., 2021). Podocyte injury undergoes the processes of podocyte hypertrophy, detachment, autophagy and apoptosis, accompanied by the reduction of podocyte marker proteins (nephrin and synaptopodin). The continuous consequences of podocyte injury destroy the renal glomerular filtration barrier, which leads to proteinuria (Nagata, 2016). Therefore, alleviation of podocyte injury is a key link to delay progression of DN.

Apoptosis is one of the mechanisms that induces podocyte injury during the progress from compensatory hypertrophy to cell detachment. Podocyte apoptosis mainly based on the emergence of apoptotic bodies along with the increased expression of pro-apoptotic protein Bax and cleaved caspase-3. It has been reported that the apoptosis rate of podocytes is significantly increased in *db/db* mice, and *in vitro*, the high glucose is sufficient to induce apoptosis in podocytes (Liu et al., 2016). Pretreatment with *Abelmoschus manihot* (TFA), significantly decreased the number of apoptotic podocytes and the expression of pro-apoptosis related proteins caspase-3 and caspase-8 in DN rats, so as to reduce proteinuria and improve renal function (Zhou et al., 2012). Therefore, inhibition of podocyte apoptosis is an essential link for relieving podocyte injury and proteinuria.

Quercetin is a polyphenol belonging to the class of flavonoids existed in bupleuri, mulberry leaves and sophora japonica et al., which is reported to have an ameliorative effect on diabetic nephropathy induced by streptozocin (Feng et al., 2019). Modern pharmacological studies show that quercetin has multiple biological functions including anti-oxidation, anti-allergic, anti-infammatory, and anti-apoptotic effects (Gomes et al., 2014; Tang et al., 2020). In addition, it has been proven to have wide pharmacological effects on diabetic diseases. Our previous studies have demonstrated that quercetin prevents renal fibrosis in DN by restraining the proliferation of mesangial cells (MCs) and the epithelial-mesenchymal transition (EMT) of renal tubular epithelial cells induced by high glucose (Lu et al., 2015; Du et al., 2019). Quercetin has a protective effect on lupus nephritis via improving the permeability of the glomerular filtration barrier to reduce proteinuria (Dos Santos et al., 2018). It suggests that quercetin has an activity of supressing proteinuria. Take it further, rutin, a precursor of quercetin, attenuates renal tubular cell apoptosis by decreasing the caspase-3/7 activities (Qu et al., 2019). It shows that quercetin has an inhibitory effect on renal cell apoptosis. Quercetin may have potential activities in relieving podocyte injury and proteinuria through inhibition of podocyte apoptosis, however, it has not yet been fully understood.

Quercetin has potential activities on resisting diabetic nephropathy, but the mechanism of action is unclear. Network pharmacology is an emerging method based on the “disease-genes-drug” network, which can be used to predict the potential mechanism of active ingredients in diseases (Boezio et al., 2017). The method combines the ideas of system biology with multi-directional pharmacology, and integrates the biological network with drug action network to analyze the relationship between drugs and nodes or modules in the network. It has been proved to possess a certain credibility and feasibility through several previous experiments (Li and Zhang, 2013; Guo et al., 2020). In this research, the target databases of quercetin and diabetic nephropathy were constructed respectively, then took intersection of the two databases, and a total of 56 possible targets for quercetin in prevention of DN were obtained. Then, epidermal growth factor receptor (EGFR) was selected as our research object through a series of subsequent analysis including the protein-protein interaction (PPI) network analysis and kyoto encyclopedia of genes and genomes (KEGG) pathway enrichment analysis.

EGFR is the member of a family of receptor tyrosine kinase ErbB receptors (Chen et al., 2012; Li et al., 2021), which is widely expressed in glomeruli, proximal tubes and collecting ducts (Zhang et al., 2014). EGFR is composed of a single extracellular ligand binding domain, a transmembrane domain and a cytoplasmic domain containing a conserved protein tyrosine core (Chakraborty et al., 2014) and is activated by binding to its ligands, leading to phosphorylation of the intrinsic kinase domain then activation of the intracellular pathways (Chen et al., 2015). These pathways include the mitogen-activated protein kinase (MAPK), janus kinase (JAK) signal transducers and activators of Transcription (STAT), src kinase and phosphatidylinositol three kinase (PI3K) pathways. They are responsible for regulating cell proliferation, differentiation, and apoptosis. Previous experimental data revealed EGFR inhibition diminishs renal injury by reducing inflammation, oxidative stress, apoptosis and fibrosis both *in vivo* and *in vitro* (Skibba et al., 2016). Mice with podocyte-specific EGFR knockout showed less podocyte loss and lighter proteinuria in streptozotocin-induced Type 1 diabetes. In the cultured immortal mouse podocytes, EGFR was knocked down by transfection of specific mouse small interfering RNA sequences and found that downregulation of EGFR expression markedly attenuated the expression of cleaved caspase three and the phosphorylation of ERK in response to high-glucose exposure (Chen et al., 2015). It has been reported that quercetin potently suppresses the autophosphorylation of the EGFR in human colon carcinoma cell, and quercetin induces apoptosis via inhibition of EGFR in breast cancer cell lines (Balakrishnan et al., 2017). However, the role of quercetin in EGFR signaling pathway and podocyte apoptosis is not yet known.

The present study was designed to evaluate the effects of quercetin on the albuminuria and podocyte apoptosis in DN. Moreover, network pharmacology was applied to explore the underlying target of quercetin against DN. In addition, we determined whether quercetin attenuates podocyte apoptosis of DN through inhibiting EGFR signaling pathway.

## Materials and Methods

### Cell Culture

Conditionally immortalized mouse podocytes (BLUEFBIO Biotechnology Company, Shanghai, China, ATCC number BFN60700330) were cultured in RPMI 1640 medium. Cells were grown in a 5% CO_2_ humidified atmosphere at 33 °C and medium containing 100 U/ml IFNγ and 10% fetal bovine serum (FBS). Then podocytes were exposed to 37°C without IFNγ for 10 days to induce differentiation. The differentiated podocytes were grown in serum-free RPMI 1640 medium for 24 h prior to the experiment, which followed by treatment with 0.1% DMSO (solvent control DMSO) or glucose (HG, 40 mM, G7021, Sigma) or quercetin (5280343, Sigma, St. Louis, MO, United States) at a concentration of 10 μmol/L (Q10), 20 μmol/L (Q20), 40 μmol/L (Q40) or 1 μmol/L AG1478 (2934816, EMD Millipore, DE) for 24 h.

### Animal Experiments

Eight-week-old genetically diabetic C57BL/KSJ *db/db* mice and their age-matched nondiabetic C57/KSJ *db/m* littermates (used as control animals) were obtained from the Model Animal Research Center of Nanjing University, following the Guiding Principles for Care and Use of Laboratory Animals of Xuzhou Medical University. All mice used in experiments were male. The mice were housed in an animal facility conditioned with 12–12 h light-dark cycles and allowed free access to normal food and water. After acclimatization for 8 weeks, the mice were randomly divided into five groups with at least six mice in each group. The average initial body weight of each group was not significantly different (*p* > 0.05). These treatment groups were designated as *db/m* group (control group), *db/db* group (diabetes model group), QL group (low-dose quercetin-treated group, 50 mg kg^−1^), QM group (medium-dose quercetin-treated group, 100 mg kg^−1^), and QH group (high-dose quercetin-treated group, 150 mg kg^−1^). Quercetin was dissolved in 0.5% carboxy methyl cellulose (CMC-Na) according to the expected dose of the treatment groups and was given to the mice via intragastric administration, whereas the mice of the *db/m* group and *db/db* group were given 0.5% CMC-Na through the same administration method for 8 weeks. After administration for 8 weeks, the mice were sacrificed and part of the kidney tissue was fixed in 4% paraformaldehyde, while the remaining tissue was stored at –80°C for biochemical analysis. Animal experiments were conducted in accordance to the principles provided by the National Institutes of Health’s Guide for the Care and Use of Laboratory Animals. Experiments were conducted with approval of the Animal Ethics Committee of Xuzhou Medical University, which also conformed the Guidelines for Ethical Conduct in the Care and Use of Animals.

### Network Pharmacology-Based Prediction of the Potential Actions of Quercetin on DN

Network pharmacology was carried out to identify the interactions between compounds and network target proteins. The putative quercetin targets were screened from ETCM (http://www.tcmip.cn/ETCM/), SymMap (http://www.symmap.org/), TCM-MESH (http://mesh.tcm.microbioinformatics.org/), TCMSP (https://old.tcmsp-e.com/tcmsp.php) four databases. Similarly, information on DN-associated target genes were gathered from the following databases: DrugBank (https://www.drugbank.ca/), OMIM (https://www.omim.org/), DisGeNet (http://www.disgenet.org/), Proteomics, then the possible targets of quercetin against DN were screened through the overlap analysis between putative targets of quercetin and known DN-associated targets. The potential target gene in quercetin was mapped to the disease target gene by using the ImageGP platform, and a Venn diagram was drawn to show results. In addition, the protein-protein interaction (PPI) network analysis was constructed to determine the potential targets and inherent pathways for investigating the actions of drugs by STRING (https://string-db.org/). Then, 12 topology analysis algorithms of Cytoscape 3.2.1 were used to screen out the key targets in PPI network. Screening of significant therapeutic targets was based on the high throughput reverse molecular docking (Gao et al., 2016; Sheng et al., 2017), hub results and the tissue distribution. The database for Annotation, Visualization and Integrated Discovery (DAVID) (https://david.ncifcrf.gov/) was used to analyze the Gene Ontology (GO) function and KEGG pathway enrichment of significant therapeutic targets. Finally, the relationships between these significantly enriched pathways and DN were further validated by literature reports.

### Measurement of Renal Function

Fasting blood glucose (FBG) was measured with a glucose assay Kit (Jiancheng Bioengineering Institute, Nanjing, China). Urinary albumin and the creatinine levels were detected by ELISA kits (Lanpai Biotechnology, Shanghai, China). The UACR (mg/g) was computed as urinary albumin/urinary creatinine. Blood urea nitrogen (BUN) was measured with ELISA kits. The ELISA kits were purchased from Lanpai Biotechnology (Shanghai, China). Results are expressed as the mean ± SEM. These biochemical indices were measured for estimating the progression of DN.

### Renal Histology Analyses

Tissue sections of 4-μm thickness were prepared from paraffin-embedded kidney tissue. The sections were stained with periodic acid Schiff (PAS) (Liu et al., 2018), periodic acid-silver metheramine (PASM), Masson, and Sirius red after deparaffinization. Staining was conducted to assess kidney morphology, the glomerular basement membrane, glycogen deposition, and collagen accumulation. These kits were purchased from Solarbio, Beijing, China. Photographs were taken randomly and blindly under a microscope (OLYMPUS, Tokyo, Japan). Quantification of staining was performed using Image Pro Plus 6.0 and was expressed as the positive region. Representative views were shown.

### Tunel Assay

Terminal deoxynucleotidyl transferase mediated dUTP nick-end labeling (TUNEL) assay (Roche, United States) is used to detect the nuclear DNA fragmentation of tissue cells in the early process of apoptosis. Sections were dewaxed and rehydrated according to standard protocols. After deparaffinization, sections were incubated with proteinase K working solution and prepared TUNEL reaction mixture for 15 min at 37°C. Sections were counterstained with DAPI (Beyotime Institute of Biotechnology, Nantong, China). The number of TUNEL positive cells per DAPI positive cells was used for quantitation of apoptotic cell.

### Hoechst 33342 Staining and Annexin V-FITC/Propidium Iodide Assay

Hoechst 33342 is a blue fluorescent dye that can penetrate cell membranes and is less toxic to cells. It was often used to detect apoptosis and the staining was observed by fluorescence microscope. The Annexin-V-FITC Apoptosis Detection Kit (BD Biosciences, Franklin Lakes, NJ, United States, catalog no. 556547) was used to detect apoptosis by flow cytometry. Cells were exposed to various conditions of treatment for 24 h, and they were harvested and processed according to the manufacturer’s instructions. Cells were considered viable if FITC Annexin V and PI staining were all negative; early apoptotic if FITC Annexin V staining was positive with negative PI staining; and late apoptotic or already dead if both FITC Annexin V and PI staining were positive (Li et al., 2017; Yao et al., 2020).

### Immunohistochemistry

The fixed kidney section of a 4 μm thickness (Leica Company, Germany, RM2235) were deparaffinized in xylene 3333 rehydrated in a graded series of alcohols. Subsequently the sections were placed in 3% H_2_O_2_ for 10 min to eliminate endogenous peroxidase activity. After pepsin antigen retrieval for 30 min, the sections were washed with PBS 3 times for 3 min each time and then blocked with 2% BSA for 0.5 h at room temperature. The sections were incubated with mouse anti-synaptopodin antibody (1:200, santa cruz biotechnology, sc-515842) at 37°C for 2 h or 4°C overnight. The sections were stained using a polymer HRP detection system (ZSGB-BIO, Beijing, China) and visualized with a DAB detection kit (Vector Laboratories Inc., Burlingame, CA, United States). After conventional dewatering and neutral balsam mounting, photographs were blindly taken at random felds under an Olympus BX43F fuorescence microscope (OLYMPUS, Japan) (Chen et al., 2019).

### Immunofluorescence

Differentiated podocytes were fixed with cold methanol at −20°C for 20 min and permeabilized with 0.1% Triton X-100/PBS. After they were washed three times with cold PBS. The cells were blocked with 2% bovine serum albumin (BSA) for 1 h at room temperature and incubated with mouse anti-synaptopodin antibody (1:200, santa cruz biotechnology, sc-515842) or rabbit anti-cleaved caspase three antibody (1:400 Cell Signaling Technology, 9661) at 37°C for 2 h or 4°C overnight. Then, the cells were washed three times with PBS and incubated with a secondary antibody conjugated to DyLight 488 or DyLight 594 (Earthox, Millbrae, CA, United States) at 37°C for 1 h, respectively. Nuclei were counterstained with DAPI. The coverslips were mounted onto glass slides, and the images were viewed with an Olympus BX43F fluorescence microscope (OLYMPUS, Japan).

### Western Blot

Protein analysis was performed on mouse renal cortex tissues (Ji et al., 2017) and cultured podocytes as described previously. EGFR, p-EGFR and cleaved caspase three antibodies were purchased from Cell Signaling (Beverly, MA, United States). ERK1/2, p-ERK1/2, Bcl2, Bax and synaptopodin antibodies were purchased from Abcam (Cambridge, United Kingdom). *β*-actin antibody was purchased from Santa Cruz Biotechnology (Santa Cruz, CA, United States).

### Statistical Analysis

All data were presented as the means ± SEM. Statistical analysis was performed using SPSS software, version 16.0 (SPSS, Inc., Chicago, IL, United States). Statistical differences were determined using analysis of variance followed by Dunnett’s test (Exp. versus Con.) using one trial analysis. A significant difference was defined as *p* < 0.05 compared with the control.

## Results

### Quercetin Reversed the Alterations in Renal Functional Parameters in Diabetic Mice

The renal function levels of the experimental mice were examined after treatment with quercetin for 8 weeks. BUN and UACR levels are important indicators of renal function. The levels of BUN and UACR in diabetic mice treated with quercetin were significantly decreased, compared with those in the diabetic mice. In parallel, the FBG levels of the diabetic mice were obviously retrieved. During the period of the study, quercetin effectively prevented the progression of albuminuria as shown in Table 1. Collectively, these results indicated quercetin exerts protective effects on renal function.

**TABLE 1:** Influence of quercetin on general parameters in experimental animals.

Groups	FBG (mmol/L)	BUN (mmol/L)	UACR (mg/g)
*db/m*	6.64 ± 0.65	5.85 ± 1.11	6.91 ± 0.13
*db/db*	40.74 ± 1.36^##^	10.56 ± 1.01^##^	45.98 ± 4.84^##^
*db/db* + QL	27.9 ± 1.74^**^	10.17 ± 1.42	39.56 ± 0.58^**^
*db/db* + QM	33.28 ± 3.29^**^	7.38 ± 0.58^**^	24.22 ± 3.39^**^
*db/db* + QH	32.1 ± 2.37^**^	5.05 ± 0.39^**^	20.44 ± 3.27^**^

Values represented mean ± SEM (n = 5/6 per group). *db/m*, normal control; *db/db*, diabetic; QL, low dose quercetin (50 mg/kg/d); QM, medium dose quercetin (100 mg/kg/d); QH, high dose quercetin (150 mg/kg/d); FBG, fasting blood glucose; BUN, blood urea nitrogen; UACR, urinary albumin creatinine ratio. ^*#*^ *p* < 0.05 vs *db/m*. ^*##*^ *p* < 0.01 vs *db/m*. ^*^ *p* < 0.05 vs *db/db*. ^**^ *p* < 0.01 vs *db/db*.

### Effects of Quercetin on Glomerular Injury in Diabetic Mice

**FIGURE 1:**
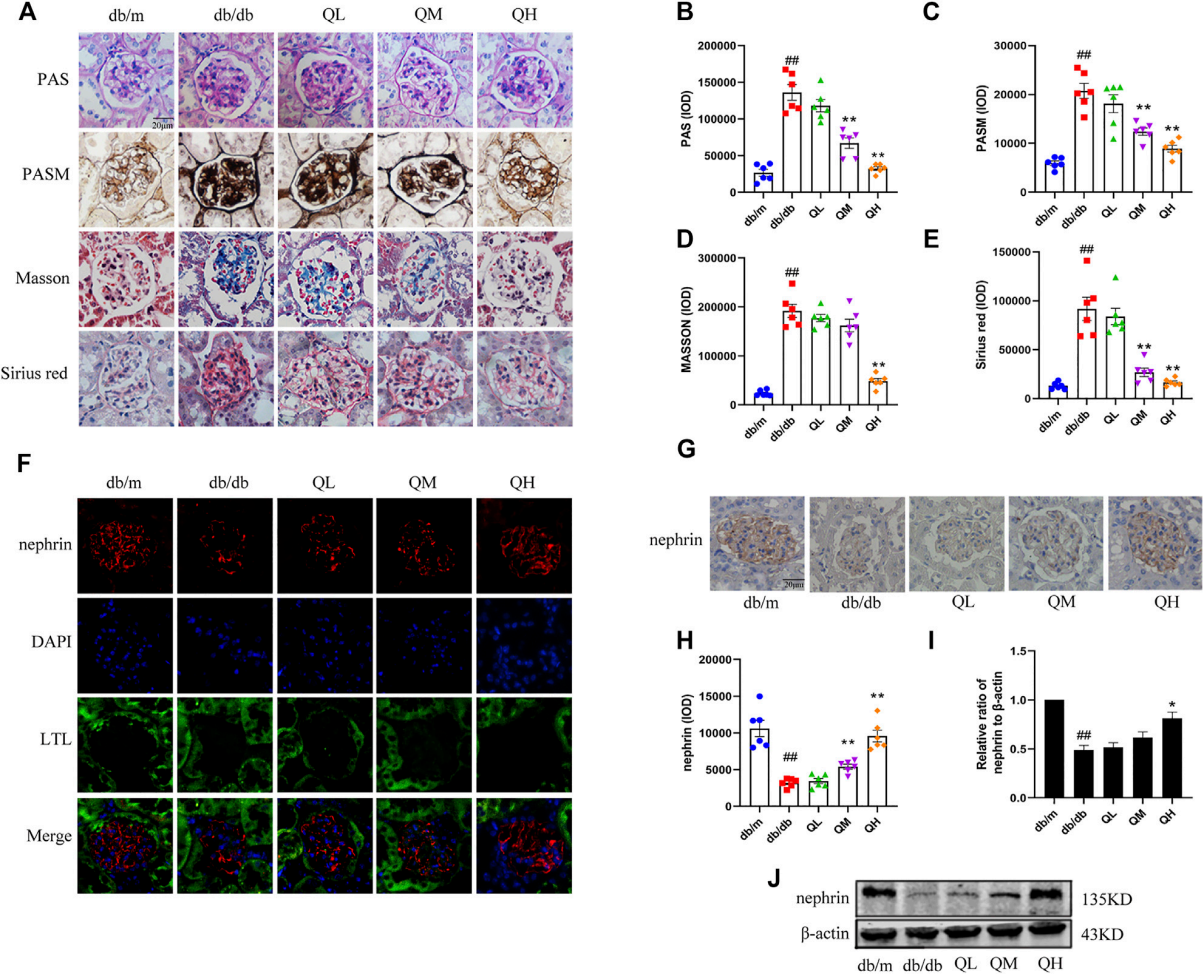
Effects of quercetin on glomerular injury in diabetic mice. **(A)** PAS, PASM, Sirius red and Masson staining of renal cortex sections in diabetic mice. **(B–E)** Statistical analysis of PAS, PASM, Sirius red and Masson staining. **(F)** Distribution and expression of nephrin through immunofluorescence with specific antibodies against podocyte marker nephrin (red), renal tubular marker LTL (green) and DAPI (blue). **(G)** Immunohistochemical analysis of nephrin expression in the five groups. **(H,I)** Statistical analysis of nephrin expression. **(J)** Expression of nephrin through Western blotting. Data were expressed as mean ± SEM, n = 6. ^*#*^ *p* < 0.05 vs *db/m*. ^*##*^ *p* < 0.01 vs *db/m*. ^*^ *p* < 0.05 vs *db/db*. ^**^ *p* < 0.01 vs *db/db*.

To investigate the protection of quercetin toward DN, a series of staining methods was used in this study. PASM staining is an essential adjunct to the evaluation of change in the GBM. The GBM was markedly thickened in the diabetic group compared with the normal group when observed by PASM stain. Glycogen and collagen are components of the extracellular matrix. PAS, masson and sirius redstaining showed that glycogen and collagen deposition was enhanced in the diabetic mice renal cortex compared with control group, however, administration of quercetin in medium-dose and high-dose effectively reversed these levels (Figures 1A–E). We quantified expression levels of a podocyte marker protein, nephrin, indicated much less nephrin expression in diabetic mice by immunofluorescence (Figure 1F), immunohistochemistry (Figures 1G,H) and western blotting analysis (Figure 1I,J). However, treatment with quercetin significantly prevented reduction of nephrin. These results indicated that quercetin protects against glomerular injury in diabetic mice.

### Quercetin Prevented Glomerular Podocyte Apoptosis in Diabetic Mice

**FIGURE 2:**
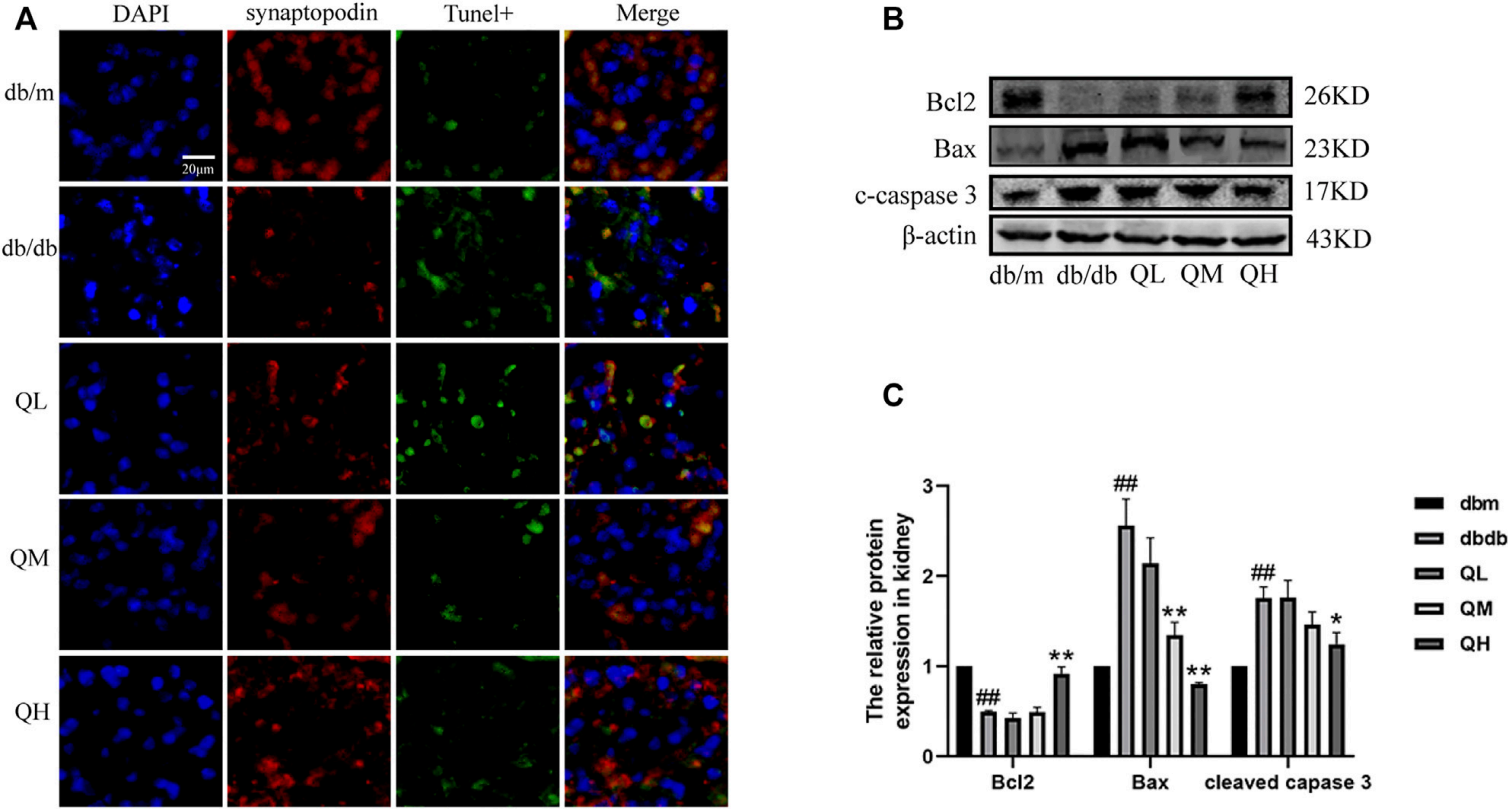
Effects of quercetin on podocyte apoptosis in diabetic mice. **(A)** Apoptosis was assessed by TUNEL assay. **(B)** Expression of Bax, Bcl-2, cleaved caspase-3 through Western blotting. **(C)** Statistical analysis of Bax, Bcl-2, cleaved caspase-3 protein expression. Data were expressed as mean ± SEM, n = 6. ^*#*^ *p* < 0.05 vs *db/m*. ^*##*^ *p* < 0.01 vs *db/m*. ^*^ *p* < 0.05 vs *db/db*. ^**^ *p* < 0.01 vs *db/db*.

TUNEL assay was used to detect apoptosis in glomeruli for observing the effect of quercetin on podocyte apoptosis under diabetic conditions. The number of apoptotic cells in glomeruli was noticeably increased from diabetic mice compared to the control group (Figure 2A, B. Further results showed that the pro-apoptotic proteins Bax and cleaved Caspase-3 were significantly up-regulated and the anti-apoptotic protein Bcl-2 was markedly down-regulated in diabetic mice (Figure 2C, D). Above changes were reversed by the administration of quercetin, which demonstrated that quercetin inhibits apoptosis in diabetic mice.

### Identification of EGFR Act as the Possible Target of Quercetin in the Prevention of Diabetic Nephropathy by Network Pharmacology Analysis

**FIGURE 3:**
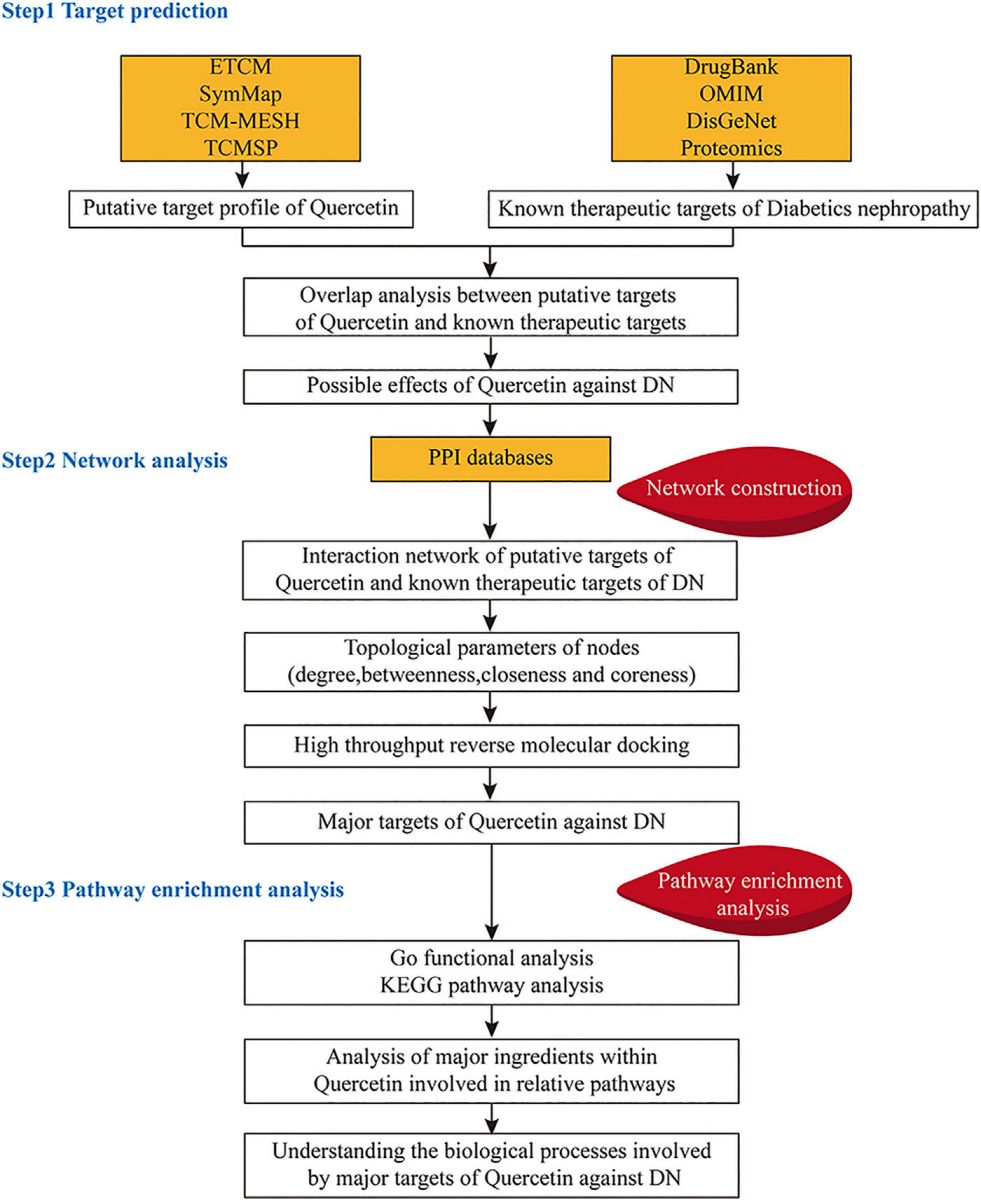
The whole framework based on an integration strategy of network pharmacology.

**FIGURE 4:**
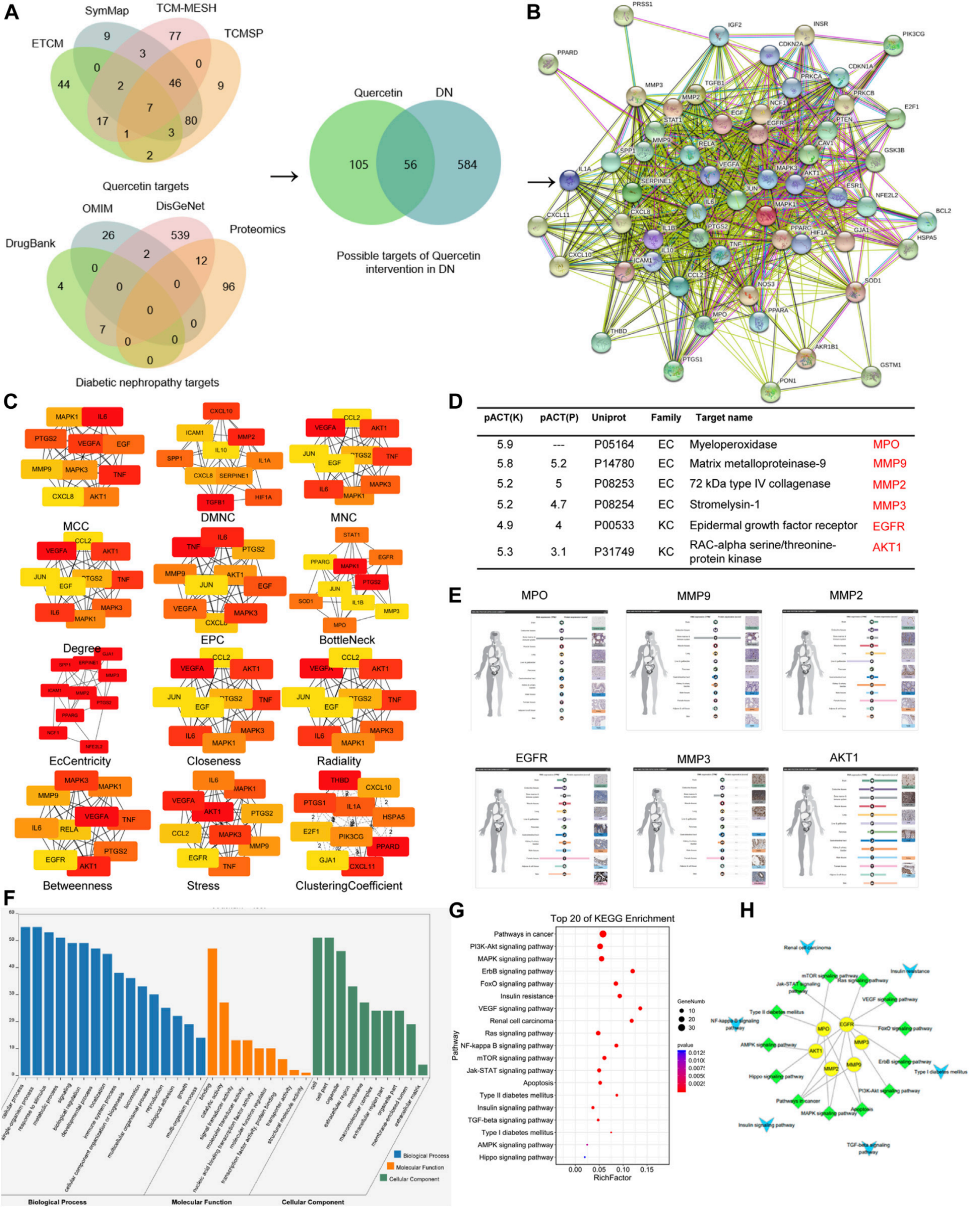
EGFR was screened as the target of quercetin in DN through network pharmacology. **(A)** Matching of target genes between DN and quercetin. **(B)** Common target PPI network between DN and quercetin. **(C)** Hub genetic analysis. **(D)** Based on the high throughput reverse molecular docking and hub results. **(E)** The distribution of tissue. **(F)** Enriched GO terms for biological process (BP) of potential targets of quercetin. **(G**,**H)** KEGG pathway analysis of putative target genes of quercetin.

We obtained 161 drugs targets and 640 DN-related disease protein targets from respective databases. A total of 56 potential targets were gained based on the intersection of protein targets acting on quercetin and these are related to DN by using the ImageGP platform. The targets were exhibited in PPI and the key targets were used to screen out through 12 topology analysis algorithms of Cytoscape 3.2.1. The six key targets MPO, MMP9, MMP2, MMP3, EGFR, AKT1 were screened in this process, which suggested that these six targets probably served as significant therapeutic targets in DN. Then the tissue distribution of these targets indicating that EGFR, MMP2, and AKT1 were expressed in the kidney. GO analysis revealed that the functions of these potential targets are related to cell proliferation, differentiation and apoptosis. In the last step, we found that EGFR enriched the most pathways by KEGG pathway enrichment analysis. Therefore, EGFR was choosed as the target for quercetin intervention in DN, which synthesized all of the above analysis. All were shown in Figures 3, 4. In addition, we evaluated the interaction between quercetin and EGFR by molecular docking scores (Supplementary Figure S1). The result showed that the docking score was -8.396, indicating a better combination of quercetin with EGFR.

### Quercetin Inhibited the EGFR Pathway in Diabetic Mice

**FIGURE 5:**
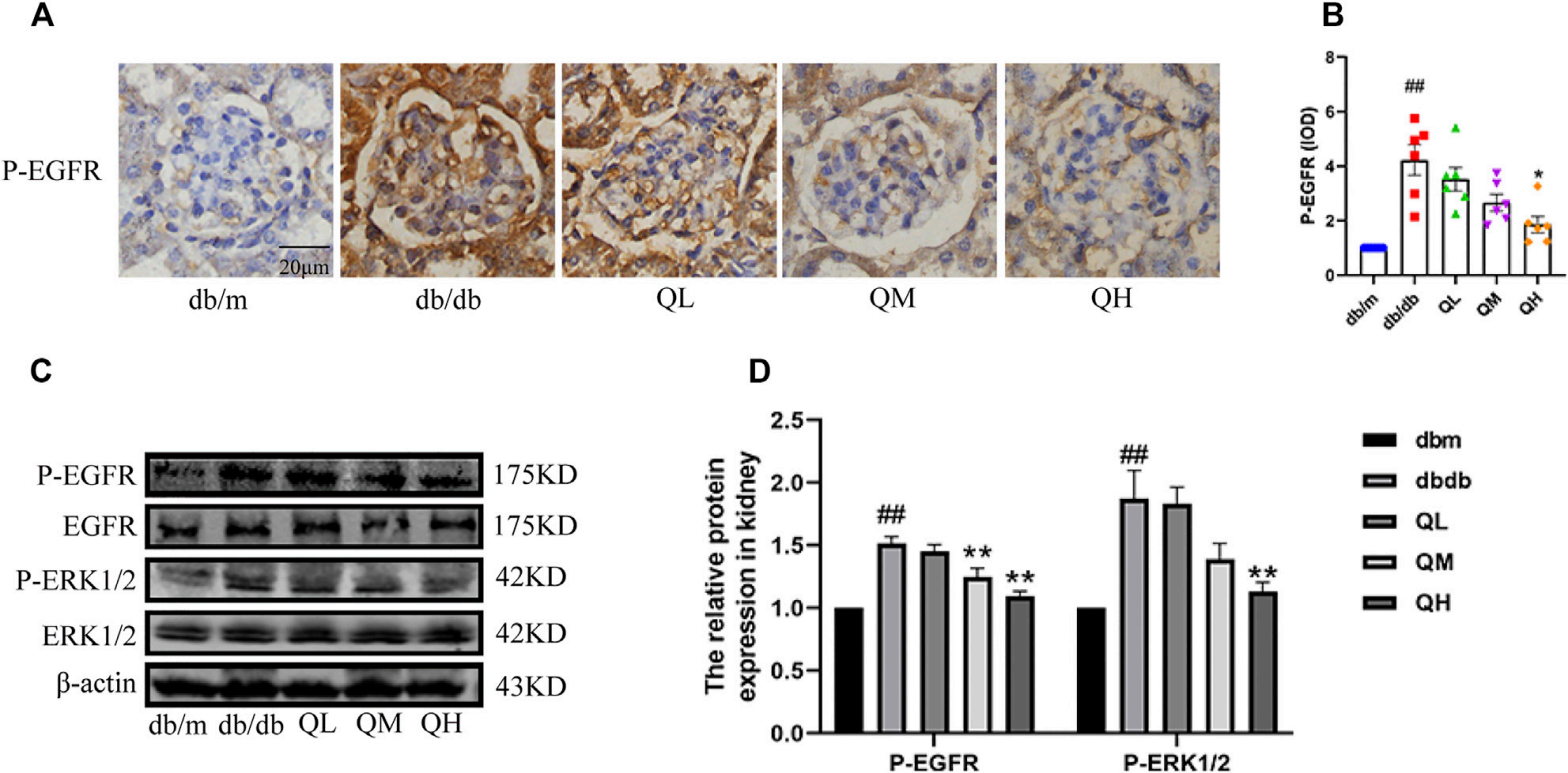
Effects of quercetin on the EGFR pathway in diabetic mice. **(A)** Immunohistochemical analysis of P-EGFR (1068) expression in the five groups. **(B)** Statistical analysis of P-EGFR (1068) expression. **(C)** Phosphorylation of EGFR and ERK in renal cortex of diabetic mice through western blotting. **(D)** Statistical analysis of PEGFR/EGFR and PERK/ERK expression. Data were expressed as mean ± SEM, n = 6. ^*#*^ *p* < 0.05 vs *db/m*. ^*##*^ *p* < 0.01 vs *db/m*. ^*^ *p* < 0.05 vs *db/db*. ^**^ *p* < 0.01 vs *db/db*.

The expressions of total EGFR, phospho-EGFR and the downstream ERK in this pathway were examined to observe whether the EGFR signaling pathway can be activated in diabetic mice. Even though the raise in total EGFR and ERK expression did not reach statistical significance, the expression of phospho-EGFR and phospho-ERK markedly increased in diabetic mice compared with the normal group, and immunohistochemical results showed the same changes Figure 5A, C), suggesting EGFR was activated under diabetic conditions and quercetin could down-regulate the expressions of phospho-EGFR and phospho-ERK in diabetic mice (Figure 5B, D). Collectively, the data demonstrated that quercetin inhibited the EGFR pathway.

### Effects of Quercetin and AG1478 on EGFR Pathway in HG-Induced Podocytes

**FIGURE 6:**
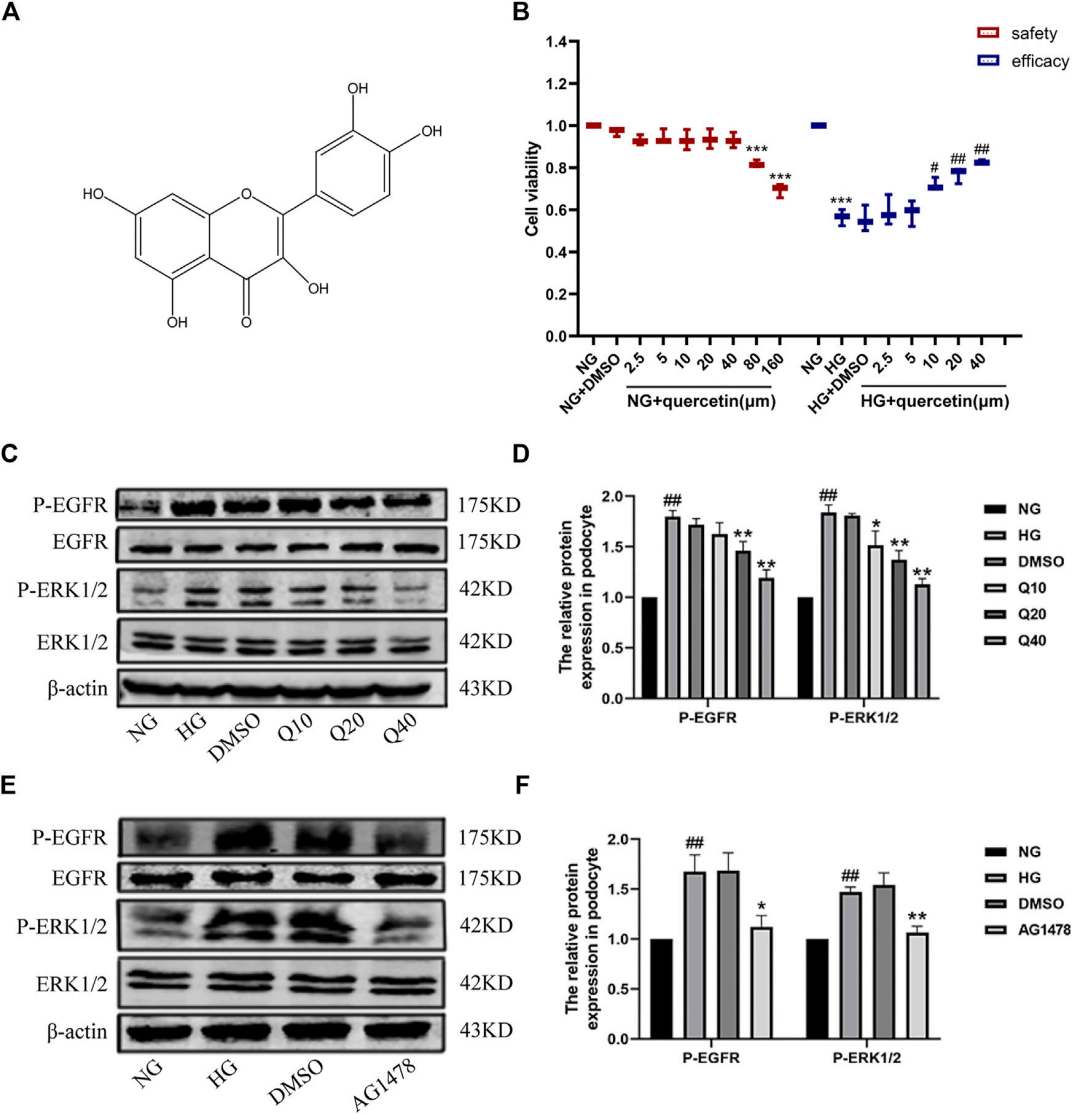
Effects of quercetin on the EGFR pathway in HG-induced podocyte. **(A)** The chemical structure of quercetin. **(B)** The safety and efficacy experiments of quercetin. **(C**,**E)** Expressions of PEGFR/EGFR and PERK/ERK through western blotting. **(D**,**F)** Statistical analysis of PEGFR/EGFR and PERK/ERK protein expression. Cells were starved for 24 h and treated with normal glucose, high glucose, DMSO, quercetin or AG1478 for 24 h. Data were expressed as mean ± SEM, n = 3. ^*##*^ *p* < 0.01 vs NG. ^*#*^ *p* < 0.05 vs NG; ^*^ *p* < 0.05 vs HG. ^**^ *p* < 0.01 vs HG.

*In vitro*, we evaluated the role of EGFR signaling through examining the expressions of phospho-EGFR and total EGFR and the downstream ERK in cultured podocytes, which were treated with HG, quercetin (40 μM) and AG1478 (EGFR inhibitor). DMSO was used as a control group for drug solvents and played no effect on their expression. CCK8 assay was used to investigate the cytotoxicity of the quercetin in podocyte. The data demonstrated that 40 μM of quercetin treatment for 24 h did not generate any conspicuous cytotoxic effects, whereas 80 μM significantly declined cell viability (Figure 6B). Then we examined cell viability under different treatments to further evaluate whether quercetin protects podocyte against HG-induced apoptosis. High glucose inhibited cell viability, while treatment with quercetin raised cell viability dose dependently. Thus, doses of 10, 20 and 40 μM quercetin were used to examine its potential ability to prevent against DN in the following experiments. Western blotting results showed that the same changes in HG-induced podocyte as *in vivo* (Figure 6C), suggesting EGFR was activated under high glucose conditions, and quercetin could down-regulate the high expressions (Figure 6D). The differentiated podocytes were treated with HG for 24 h prominently increased EGFR phosphorylation, and pretreatment of the differentiated podocytes with the EGFR tyrosine kinase inhibitor (AG1478) and HG markedly prevented EGFR (Figure 6E, F, Supplementary Figure S2). So we used AG1478 to further explore the mechanism of quercetin on DN.

### Quercetin Reversed Podocyte Apoptosis Through Inhibiting the EGFR Pathway

**FIGURE 7:**
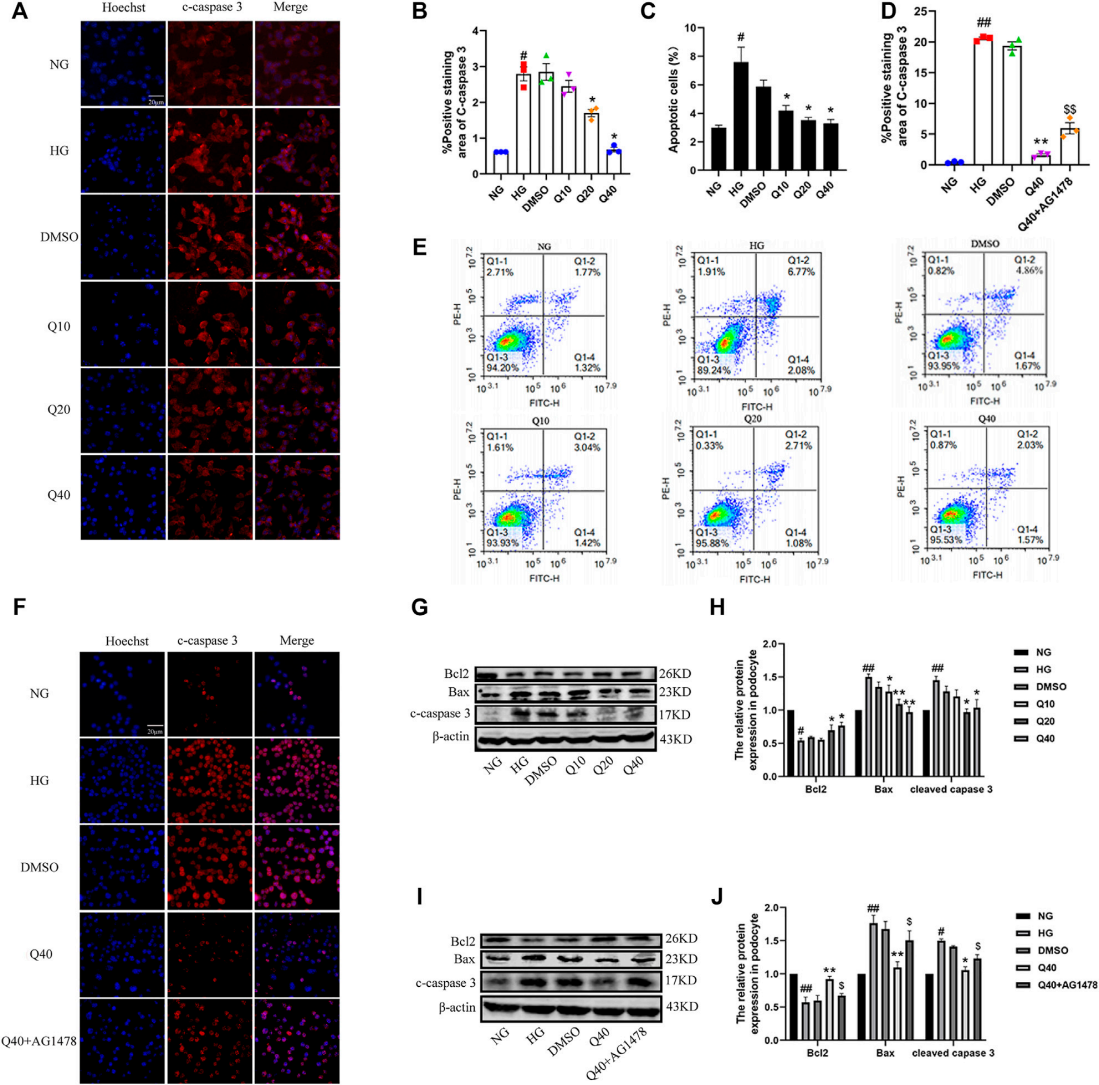
Effects of quercetin on HG-induced podocyte apoptosis through the EGFR pathway. **(A**,**F)** Distribution and expression of cleaved caspase three in podocyte through immunofluorescence, the number of apoptotic cells was assessed by Hoechst 33342 staining. **(B**,**D)** Statistical analysis of cleaved caspase three expression. **(C)** Percentage of apoptotic podocytes. **(E)** Flow cytometry analysis of podocytes apoptosis with Annexin V-FITC/PI staining. **(G**,**I)** Expression of Bcl2, Bax and cleaved caspase three through western blotting. **(H**,**J)** Statistical analysis of Bcl2, Bax and cleaved caspase three protein expression. Data were expressed as mean ± SEM, n = 3. ^*##*^ *p* < 0.01 vs NG. ^*#*^ *p* < 0.05 vs NG; ^*^ *p* < 0.05 vs HG. ^**^ *p* < 0.01 vs HG. ^*$*^ *p* < 0.05vs Q40; ^*$$*^ *p* < 0.01vs Q40.

Podocyte apoptosis was detected by Annexin V-staining (Figure 7C, E), immunofluorescence and western blot analysis. We found that quercetin reversed HG-induced podocyte apoptosis through examining the expression of Bcl-2, Bax and cleaved caspase-3 (Figure 7A, B, G, H). To further investigate whether quercetin improved HG-induced podocyte apoptosis through the EGFR pathway, we used AG1478 to inhibit EGFR and then added quercetin to observe its effect on podocyte apoptosis. Compared to Q40 group, the improvement level of podocyte apoptosis was reduced in Q40 + AG1478 group (Figure 7D, F, I, J), indicating that quercetin supressed HG-induced podocyte apoptosis through the EGFR pathway.

### Quercetin Inhibited HG-Induced Podocyte Injury Through the EGFR Pathway

**FIGURE 8:**
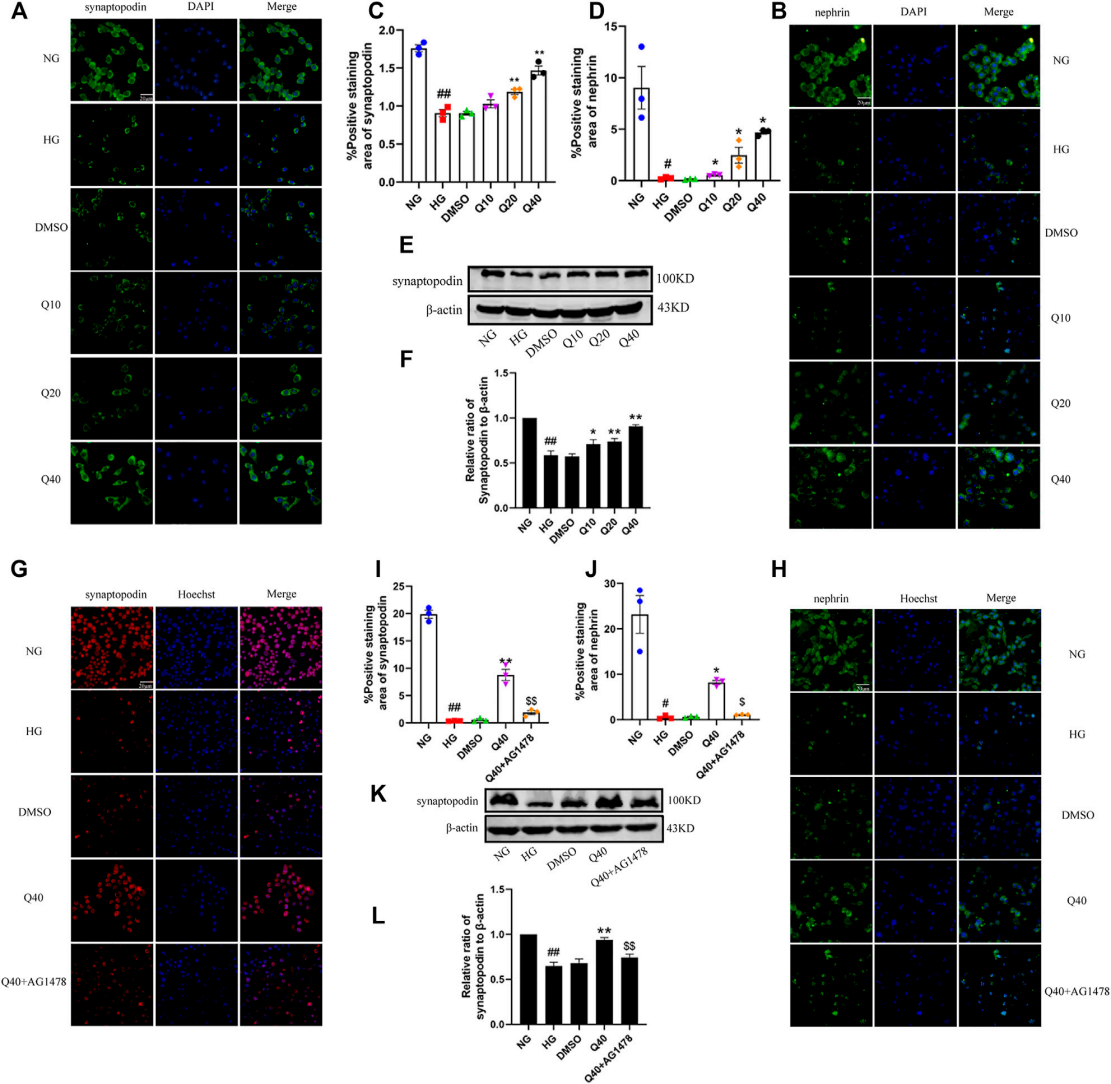
Effects of quercetin on HG-induced podocyte injury through the EGFR pathway. **(A**,**B**,**G**,**H)** Distribution and expression of synaptopodin and nephrin in podocyte through immunofluorescence. **(C**,**D**,**I**,**J)** Statistical analysis of synaptopodin, nephrin expression. **(E**,**K)** Expression of synaptopodin through western blotting. **(F**,**L)** Statistical analysis of synaptopodin protein expression. Cells were starved for 24 h and treated with normal glucose, high glucose, DMSO, quercetin, AG1478 or Q40 + AG1478 for 24 h. Data were expressed as mean ± SEM, n = 3. ^*##*^ *p* < 0.01 vs NG. ^*#*^ *p* < 0.05 vs NG; ^*^ *p* < 0.05 vs HG. ^**^ *p* < 0.01 vs HG. ^*$*^ *p* < 0.05vs Q40; ^*$$*^ *p* < 0.01vs Q40.

We identified expression levels of synaptopodin and nephrin by immunofluorescence and western blotting analysis. As shown in Figures 8A–F, quercetin raised the low expression of synaptopodin and nephrin in HG-induced podocytes, but the upregulation effect was supressed in Q40 + AG1478 group (Figure 8G–L), verifying that quercetin inhibited HG-induced podocyte injury through the EGFR pathway.

**FIGURE 9:**
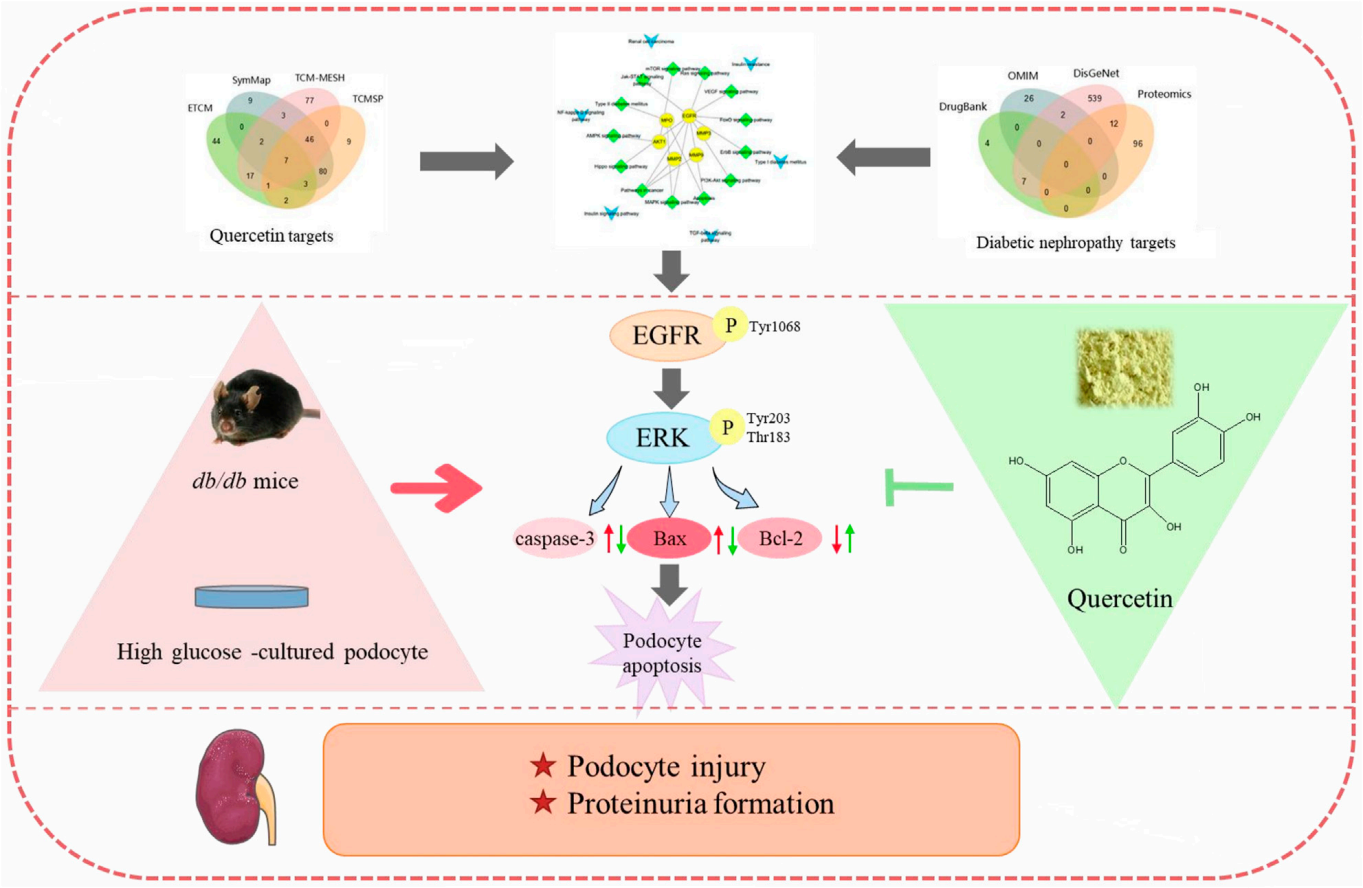
Quercetin attenuates podocyte apoptosis of diabetic nephropathy through targeting EGFR signaling. EGFR was screened from quercetin targets and diabetic nephropathy targets by network pharmacology analysis. In diabetic nephropathy, the phosphorylation rate of EGFR and ERK were raised, leading to increased podocyte apoptosis, finally resulting in podocyte injury and proteinuria formation. By restraining the activation of EGFR signaling pathway, quercetin can relieve podocyte apoptosis and ameliorate podocyte injury, thus delaying the formation of proteinuria and the progression of diabetic nephropathy.

## Discussion

In spite of DN pathogenesis has made progress, its high incidence and poor prognosis have not been greatly improved. Therefore, excavating novel therapeutic targets for DN is significant. The appearance of proteinuria is regarded as the hallmark of DN at its early stage. Podocyte hypertrophy, detachment and apoptosis can all lead to the increase of proteinuria in patients with DN. A multitude of studies has demonstrated that therapies with lessening podocyte apoptosis have beneficial effects on kidney diseases (Liu et al., 2016; Jin et al., 2019). Therefore, lessening podocyte apoptosis is considered to be a potential novel therapeutic strategy for DN. The results presented here indicate that quercetin decreases the apoptosis rate of podocytes. Considering the protective effects of quercetin in podocytes, we hypothesize that quercetin has a protective role in diabetic nephropathy by reducing podocyte apoptosis.

Currently, hyperglycemia is regarded to be the dangerous triggering factor in the progression of diabetic nephropathy (Brownlee, 2005). Administrated quercetin in diabetic mice, a significant descent of the FBG levels was encouraging. The declining FBG level, which was statistically meaningful but absent clinically practical significance, indicating that quercetin may exert a protective effect on the kidney independent from the insulin receptor-dependent pathway. Notably, quercetin remarkably improved the signals of renal damage and dysfunction that were reflected by a reduction in BUN and UACR in diabetic mice. These results are supported by a previous study that similarly showed a remarkable effect of quercetin on ameliorating the renal function in diabetic nephropathic rats (Tang et al., 2020). Exposure to a high glucose milieu induces podocyte injury and leads to the impaired filtration barrier function of glomeruli, ultimately results in proteinuria (Xu et al., 2015; Qi et al., 2017). An abundance of evidence indicates that nephrin and synaptopodin is important in podocytes both for the slit membrane structure of interpodocytes and the integrity of the filtration barrier (Ettou et al., 2020). Our results demonstrated that the expression of nephrin was markedly decreased in diabetic mice and HG-induced podocytes, whereas quercetin treatment recovered the level of nephrin. Previous data suggested a protective effect of quercetin against podocyte injury through recovering podocytes foot processes with scarce focal fusion and increasing the expression of podocyte markers podocin in the lupus nephritis mice (Dos Santos et al., 2018), which is consistent with our results. These results indicated that quercetin improves renal function and protects against glomerular podocytes injury.

During the entire cell growth and development process, apoptosis serves a crucial role in maintaining cell stability, and dysregulation of apoptosis presents a dangerous factor in various diseases (Godse et al., 2017). Previous research showed that renal cellular apoptosis and the increased expression of apoptosis-related proteins Bax and caspase-3 lead to renal tubular atrophy and interstitial fibrosis, even aggravated renal damage in human lupus nephritis (Cui et al., 2012). Wang et al. found that the cleaved caspase-3 protein levels were significantly raised and the number of podocytes was significantly decreased in diabetic rats (Wang et al., 2019). At present study, the results showed markedly up-regulated levels of Bax, cleaved caspase-3, but a noticeably down-regulated level of Bcl-2 in HG-induced podocytes and diabetic mice. However, treatment with quercetin reversed these changes, implying that quercetin can attenuate podocyte apoptosis under diabetic conditions. This result is in line with that of another experiment in which pretreatment with quercetin could have anti-apoptotic effect against lipopolysaccharide-induced osteoblast apoptosis (Guo et al., 2017).

Therefore, a better understanding of the molecular mechanisms underlying these effects is crucial. The approach of network pharmacology was regarded as the fastest and most effective screening method in the early study of drug effectiveness (Huang et al., 2020). We concluded that quercetin produces a multi-target effect in the treatment of DN by utilizing network pharmacology analysis. Here, we have only studied EGFR, the target with the largest number of enrichment pathways. Quercetin also acts on other signaling pathways possibly, including PI3K/AKT signaling pathway, MAPK signaling pathway and Hippo signaling pathway. We had previously found that quercetin inhibited EMT of renal tubular epithelial cells through PI3K/AKT signaling pathway, indicating that PI3K/AKT signaling pathway was activated in kidney. As a result, this discovery validates the value of our earlier work. In the current research, we hypothesized it was inhibition of EGFR that podocyte apoptosis was rescued by quercetin. Chen et al. reported that EGFR deletion in podocytes attenuates diabetic nephropathy (Chen et al., 2015). Another experiment revealed that inhibiting EGFR improved fibrosis and apoptosis in renal tissue by downregulating the expression of TGF-β, collagen IV and Bax, which is in accordance with ours (Skibba et al., 2016).

EGFR signaling cascade is a key regulator in cell proliferation, differentiation, division, survival, and disease development (Zhang et al., 2017). The EGFR small-molecule tyrosine kinase inhibitors (TKIs) emerge as a promising inhibitory approach targeting EGFR. AG1478 is one of the EGFR-TKIs (Zhang et al., 2008), which targets the adenosine triphosphate binding site on the intracellular kinase domain and prevents tyrosin kinase activation to inhibit EGFR (Pawara et al., 2021). In this experiment, AG1478 was used to examine the relationship between EGFR signaling pathways and quercetin-rescued podocyte apoptosis through detecting the expression of apoptosis-related proteins by co-cultured of AG1478 and quercetin. Expectedly, the observations indicated that quercetin abated the expression of Bax, cleaved caspase-3 through EGFR signaling pathway. This result is in line with that of another study in which EGFR inhibitor supressed podocyte hypertrophy, then led to the reduction of podocyte apoptosis and significantly decreased the albuminuria and glomerular enlargement (Lee et al., 2015). Consequently, quercetin, as a natural compound, is of noticeable help in the adjuvant therapy by inhibiting EGFR.

Taken together, this study provided a novel approach to reveal the therapeutic mechanisms of quercetin against DN, the experimental results demonstrated that quercetin inhibited podocytes apoptosis *in vitro* and *in vivo* by regulating the EGFR pathway. Quercetin shows great potential to be developed as a candidate drug for treating DN.

## Data Availability

The raw data supporting the conclusions of this article will be made available by the authors, without undue reservation.
