# Supplementary material for: Quercetin Attenuates Podocyte Apoptosis of Diabetic Nephropathy Through Targeting EGFR Signaling
Source: Front Pharmacol. 2022 Jan 5;12:792777. doi: 10.3389/fphar.2021.792777 (PMC8766833; doi:10.3389/fphar.2021.792777)
Supplement: Supplementary file 1 [file DataSheet1.docx]

Supplementary Material

## Supplementary Figure


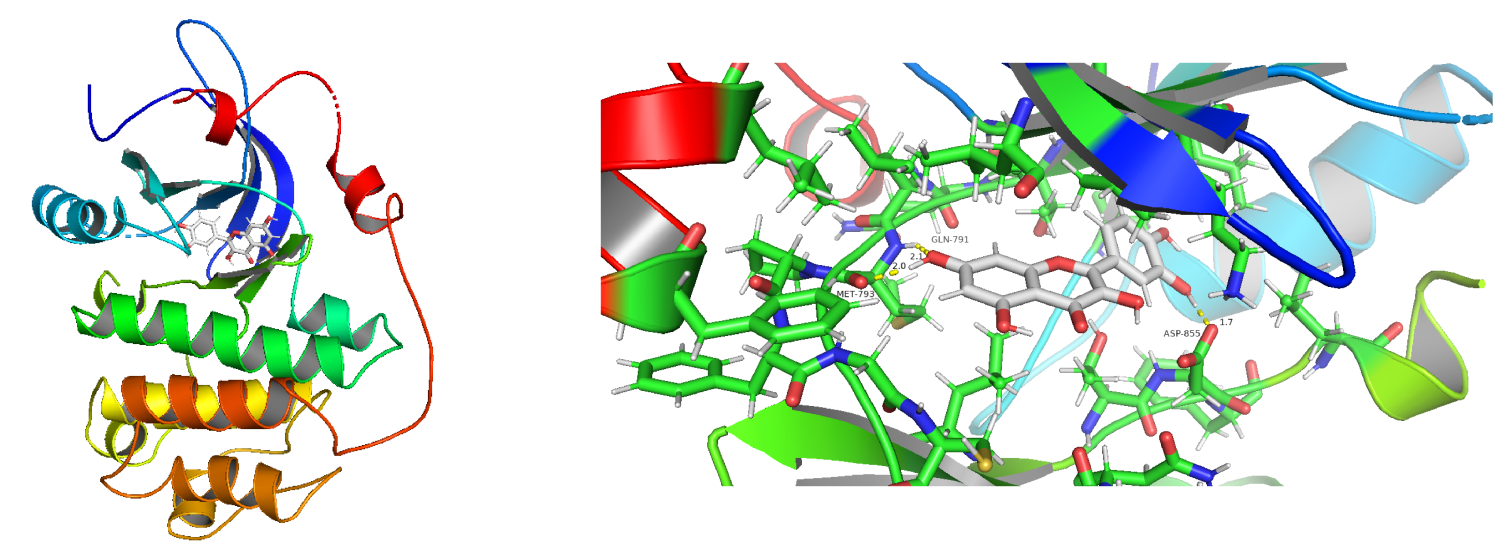


**Figure S1.** Computational docking model predicts the interaction between quercetin and wildtype EGFR. Intermolecular forces are formed between quercetin and the wildtype EGFR at Gln791, Met793 and Asp855.


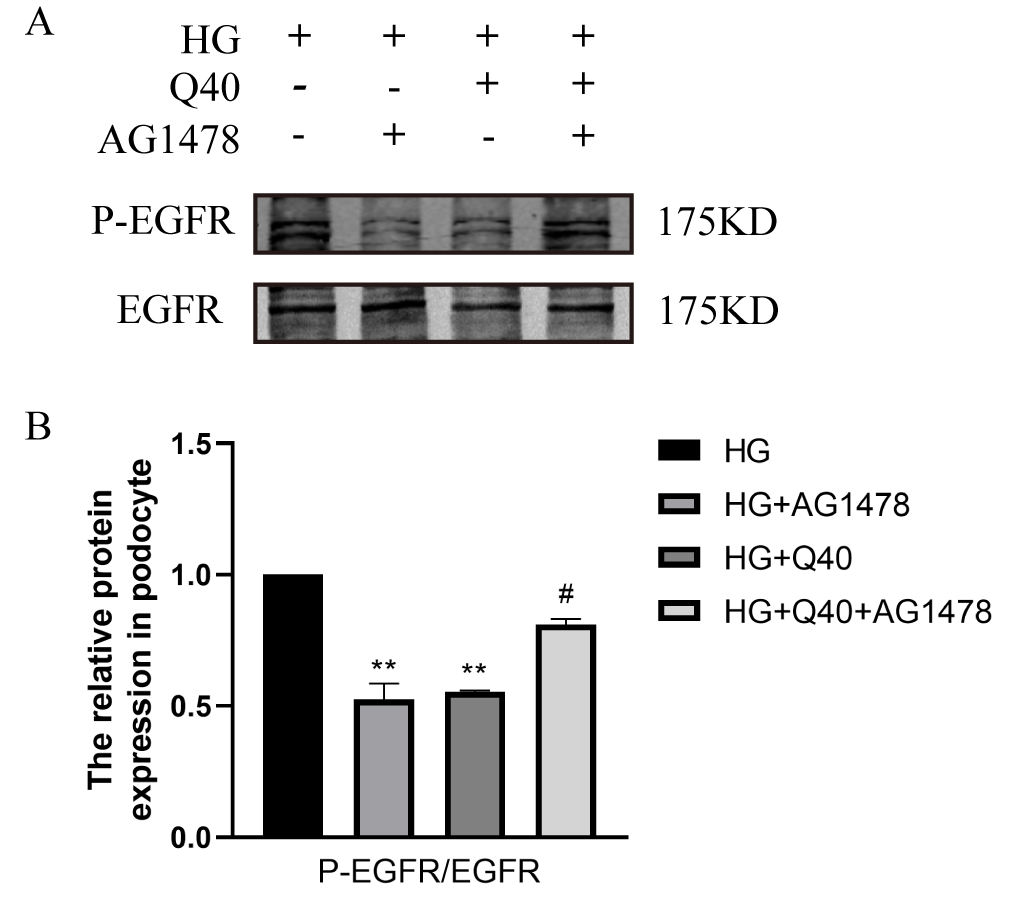


**Figure S2.** Treatment with quercetin and AG1478 inhibited EGFR pathway in HG-induced podocyte. **(A)** Expressions of PEGFR/EGFR through western blotting. **(B)** Statistical analysis of PEGFR/EGFR protein expression. Cells were starved for 24 h and treated with high glucose, quercetin or AG1478 for 24 h. Data were expressed as mean± SEM, n = 3. *^**^P*<0.01 vs HG, *^#^P*<0.05 vs HG+AG1478.
